# Compressible and monolithic microporous polymer sponges prepared *via* one-pot synthesis

**DOI:** 10.1038/srep15957

**Published:** 2015-11-04

**Authors:** Yoonbin Lim, Min Chul Cha, Ji Young Chang

**Affiliations:** 1 Department of Materials Science and Engineering, College of Engineering, Seoul National University, Seoul 151-744, South Korea

## Abstract

Compressible and monolithic microporous polymers (**MP**s) are reported. **MP**s were prepared as monoliths *via* a Sonogashira–Hagihara coupling reaction of 1,3,5-triethynylbenzene (**TEB**) with the bis(bromothiophene) monomer (**PBT-Br**). The polymers were reversibly compressible, and were easily cut into any form using a knife. Microscopy studies on the **MP**s revealed that the polymers had tubular microstructures, resembling those often found in marine sponges. Under compression, elastic buckling of the tube bundles was observed using an optical microscope. **MP-0.8**, which was synthesized using a 0.8:1 molar ratio of **PBT-Br** to **TEB**, showed microporosity with a BET surface area as high as 463 m^2^g^–1^. The polymer was very hydrophobic, with a water contact angle of 145° and absorbed 7–17 times its own weight of organic liquids. The absorbates were released by simple compression, allowing recyclable use of the polymer. **MP**s are potential precursors of structured carbon materials; for example, a partially graphitic material was obtained by pyrolysis of **MP-0.8**, which showed a similar tubular structure to that of **MP-0.8**.

The fascinating morphologies of sea sponges have inspired researchers in materials science. The outer surface cells of a sponge have many small holes called dermal pores through which large volumes of water can move inside the sponge. Internal channels are also found in the outer surface cells. The skeleton of a sponge consists of collagens and inorganic components, such as silica and calcium carbonate. Some sponges have collagen fibres that constitute a network structure, called spongin^1^. Sponge-like structures have been widely adapted for synthetic materials and have shown enhanced performance in their application in various areas, such as nanogeneration, catalysis, supercapacitance, photovoltaics, drug delivery, and tissue generation^2^,^3^,^4^,^5^,^6^,^7^,^8^.

As a material, a sponge is characterized by its porosity, flexibility, and compressibility. Sponges with a network structure of spongin fibres obtained from sea animals have been used for removing liquids by absorption since ancient times. There is also a variety of synthetic polymer sponges available. One of the most common methods to synthesize sponges is to mix a polymer with an inorganic crystal, such as sodium sulfate, which is then removed by heating the mixture to generate pores in the polymer matrix. The pore size formed depends on the size of the crystals, which usually ranges from the millimetre down to the micrometre scale. Sponges prepared using the emulsion solvent diffusion method are known to have pores in the mesopore range^9^. Polymer sponges based on materials, such as chitosan^10^, melamine^11^, cellulose^12^, and polydimethylsiloxane^13^ have been chemically modified to provide them with properties suitable for specific applications.

Recently, there have been efforts to synthesize sponges with small pores using a bottom-up approach. Gui *et al.* reported on monolithic carbon nanotube (CNT)-based sponges using a chemical vapour deposition process employing ferrocene and 1,2-dichlorobenzene as a catalyst precursor and a carbon source, respectively^14^. These CNT sponges had a surface area of 300–400 m^2^g^–1^ and an average pore size of about 80 nm. Hashim *et al.* synthesized macroporous (pore diameter >50 nm) CNT sponges *via* a boron-doping strategy during the chemical vapour deposition of toluene using ferrocene as the catalyst precursor. Excess boron atoms were found in the “elbow” junctions forming nanotube covalent interconnections^15^.

The most attractive feature of sponges is their compressibility, which enables easy removal of absorbates by applying pressure. While sponges with large pores are mainly used for removing liquids by absorption, sponges with micropores will have a wider range of applications, such as in molecular storage, separation, and catalysis. According to IUPAC notation^16^,^17^, microporosity refers to porosity with pores having a diameter <2 nm. Zeolites, activated carbons, and metal–organic frameworks (MOFs) are typical microporous materials, but they are not compressible. Recently, microporous organic polymers (MOPs) have been studied extensively because of their versatile functionality and mechanical stability. Most MOPs are usually prepared using a stepwise polymerization of tri- or higher multifunctional building blocks, and are obtained as precipitated particles because of their cross-linked structures. Although macroscopic gels^18^,^19^ or monolithic polymers^20^,^21^,^22^ are sometimes formed, they are easily broken into pieces after drying. To our knowledge, compressible microporous polymers have not yet been reported.

Herein, we report on compressible, monolithic microporous polymer sponges with tubular structures, prepared *via* a one-pot synthesis. We synthesized a series of microporous polymers using the cross-coupling reactions of multifunctional aromatic monomers. From among several possible combinations, the reaction conditions were clarified under which compressible polymer monoliths could be produced. In this paper, we discuss the compressibility mechanisms along with the microstructures and porous properties of these polymers.

## Results

### Synthesis and characterization

**Figure 1:**
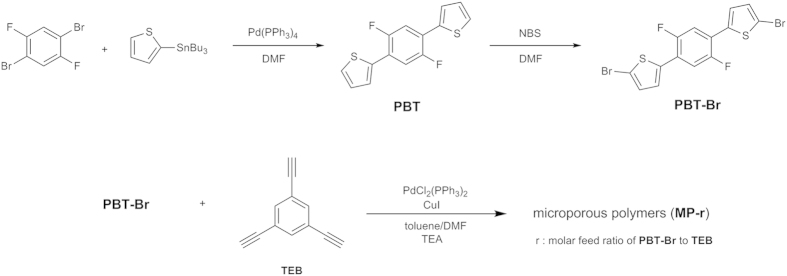
Reaction scheme for the synthesis of the bis(bromothiophene) monomer (PBT-Br) and microporous polymers (MPs). The polymers were prepared by a Sonogashira-Hagihara coupling reaction.

Compressible **MP**s were prepared *via* a Sonogashira–Hagihara coupling reaction of 1,3,5-triethynylbenzene (**TEB**) with the bis(bromothiophene) monomer (**PBT-Br**) (Fig. 1)^18^. **PBT-Br** was synthesized from a Stille coupling reaction of 1,4-dibromo-2,5-difluorobenzene with 2-(tributylstannyl)thiophene, followed by bromination with *N*-bromosuccinimide. The polymerization reactions of **PBT-Br** and **TEB** were carried out in a mixture of toluene and *N*,*N*-dimethylformamide (DMF) (1:1, v/v). The **TEB** dissolved well in the polymerization medium at room temperature, but the **PBT-Br** was only slightly soluble. Unless otherwise noted, the polymerization reactions were carried out at 100 °C for 24 h. The monomer feed ratio influenced the resulting polymer morphology. Five polymers were synthesized, **MP-0.5**, **MP-0.8**, **MP-1.0**, **MP-1.2**, and **MP-1.5**, which were synthesized by changing the molar ratio between **PBT-Br** and **TEB** as 0.5:1, 0.8:1, 1:1, 1.2:1, and 1.5:1, respectively. **MP-0.8**, **MP-1.0**, **MP-1.2**, and **MP-1.5** were obtained as monoliths, but **MP-0.5** was obtained as a powder.

2,2′-(1,4-Phenylene)bis(5-bromothiophene) is an structural analog of **PBT-Br** and has no fluoro groups. The polymerization reaction of the analog with **TEB** (the molar ratio between the analog and **TEB** = 0.8:1) in toluene/DMF (1:1 v/v) gave only powders, whereas in toluene, the same reaction produced a compressible monolith consisting of tubes and flakes (Supplementary Fig. S1). 2,2′-(1,4-Phenylene)bis(5-bromothiophene) was less soluble in toluene compared with **PBT-Br** and practically insoluble in DMF at room temperature. These results showed that the appropriate solubility of the reactants was also important for the formation of a compressible monolithic polymer.

It was very notable that cross-linked microporous polymers were formed as monoliths, which were usually obtained as powders^23^,^24^,^25^,^26^,^27^,^28^. Although some studies have reported where the reaction mixture turned into a gel during the Sonogashira–Hagihara coupling reactions of multifunctional monomers, the gel then fragmented into pieces when applying workup processes^18^,^19^.

**Figure 2:**
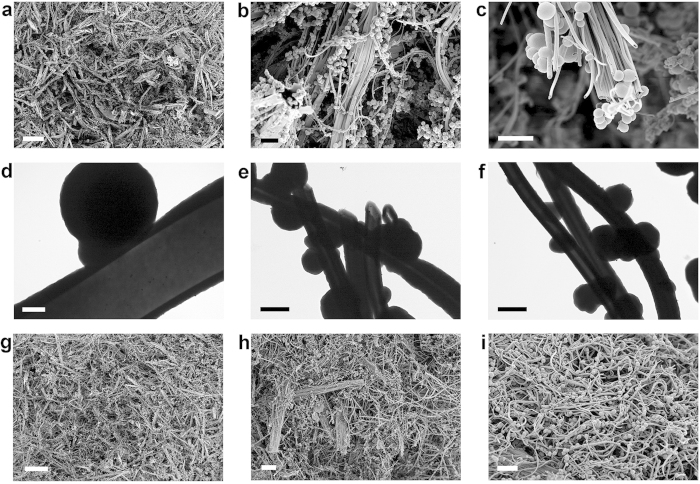
Electron microscope images of MPs. (**a–c**) SEM images of **MP-0.8** (scale bar: a = 100 μm, b = 10 μm, and c = 5 μm). (**d–f**) TEM images of **MP-0.8** (scale bar: d = 500 nm, e = 1 μm, and f = 1 μm). SEM images of (**g**) **MP-1.0** (scale bar = 100 μm), (**h**) **MP-1.2** (scale bar = 20 μm), and (**i**) **MP-1.5** (scale bar = 10 μm).

Electron microscopy studies have shown that monolithic polymers have very interesting microstructures. Figure 2a–c shows scanning electron microscope (SEM) images of **MP-0.8** that consisted mainly of tubes with diameters in the range of a few hundred nanometres, together with spherical particles on the micrometre scale. Transmission electron microscope (TEM) and SEM images suggested that these tubes had hollow structures with open ends (Fig. 2d–f). This microstructure resembles that often observed in marine sponge animals^29^. Some tubes were bundled into fibrous assemblies that had become entangled with each other to form a monolithic structure^14^. Powder X-ray diffraction (PXRD) measurements revealed that **MP-0.8** was amorphous without any long-range ordering (Supplementary Fig. S2).

Element mapping of the main elements of **MP-0.8** by energy dispersive spectroscopy (EDS) showed that carbon, fluorine, and sulfur were distributed uniformly throughout the sample (Supplementary Fig. S3). This result suggests that the Sonogashira–Hagihara cross-coupling reaction between **PBT-Br** and **TEB** monomers occurred predominantly, although a homocoupling reaction of **TEB** could occur under the reaction conditions used^19^. Relatively higher concentrations of carbon, fluorine, and sulfur in the spherical particle regions reflected the solid character of the particles and the hollow structure of the tubes, which agreed well with the results obtained from the TEM images.

The other monolithic polymers also had similar fibrous tubular microstructures. Figure 2g–i shows SEM images of **MP-1.0**, **MP-1.2**, and **MP-1.5**, respectively. Notably, more tube bundles with diameters around 10 μm were observed in **MP-0.8** and **MP-1.0** than in other polymers. **MP-1.5** consisted mainly of highly entangled individual tubes. **MP-1.0** and **MP-1.2** showed a compressible behaviour that was comparable to **MP-0.8**, but **MP-1.5** was hard with low compressibility.

The porosity of **MP**s was investigated using cryogenic N_2_ adsorption–desorption experiments (Supplementary Fig. S4). The gradient of the N_2_ adsorption isotherm increased sharply at low relative pressures, indicating the existence of micropores. Non-local density functional theory (NL-DFT) pore size distribution calculations on **MP**s also revealed that the polymer had micropores with diameters <2 nm along with mesopores. Because the polymer morphology was influenced by the monomer feed ratio used in the polymer synthesis, so too was the resulting polymer porosity. The BET surface areas of the polymers decreased as the molar ratio of **PBT-Br** increased, so that **MP-0.5** showed the highest BET surface area of 853 m^2^g^–1^, followed by **MP-0.8** (463 m^2^g^–1^), **MP-1.0** (109 m^2^g^–1^), **MP-1.2** (62 m^2^g^–1^), and **MP-1.5** (non-porous) (Supplementary Fig. S5). This tendency was attributable in part to the formation of a less dense network on incorporation of more **PBT-Br**. The BET surface area of **MP-0.5** was similar to that of a **TEB** homocoupled microporous polymer reported in the literature (842 m^2^g^–1^)^19^, suggesting that the homocoupling of ethynyl groups was dominant because they were in excess in the reaction mixture. The polymer obtained from 2,2′-(1,4-phenylene)bis(5-bromothiophene) and **TEB** (the molar ratio = 0.8:1) in toluene/DMF (1:1 v/v) showed the BET surface area of 86 m^2^g^−1^. We focused on **MP-0.8** for further experiments, which showed the highest surface area among the compressible monolithic polymers.

**Figure 3:**
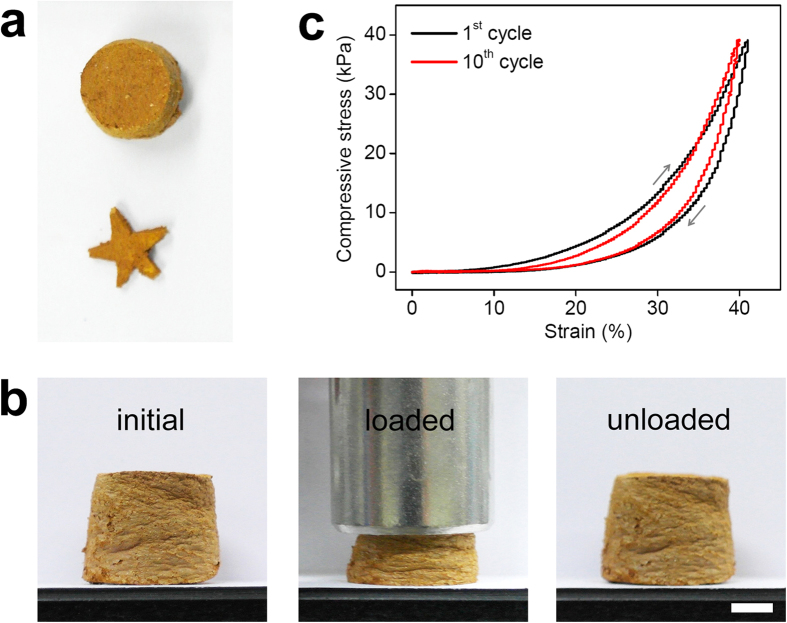
Monolithic and compressible properties of MP-0.8. (**a**) Images of **MP-0.8** prepared in a 10 mL vial as a mold (upper) and of the polymer cut into a star shape (lower). (**b**) Images of **MP-0.8** under loaded and unloaded conditions (scale bar = 5 mm). (**c**) Compressive stress–strain curves of **MP-0.8** for the first and tenth test cycles.

**MP-0.8** was prepared in various shapes and sizes that were retained, even after several workup processes, including washing, Soxhlet extraction, and drying at elevated temperatures. Figure 3a shows an image of **MP-0.8** prepared in a 10 mL vial as a mold. Dry **MP-0.8** was easily cut into any form using a knife. The polymer exhibited reversible compressibility (Fig. 3b and Supplementary Movie S1). Only a small change was observed in the compressive stress–strain curves (Fig. 3c) measured over 10 cycles of repeated stress loading and unloading, which suggested a high mechanical stability of the polymer against compressive force^11^,^30^. Hysteresis in the compressive stress-strain curve indicated the stiffness of the polymer^31^. Compressibility is usually exhibited in solids having cellular or foam structures containing pores or voids inside^32^; however, to our knowledge, observing a compressible behaviour in highly cross-linked microporous polymers is unprecedented.

The solid state ^13^C cross-polarization/magic-angle spinning nuclear magnetic resonance (CP/MAS NMR) spectrum of **MP-0.8** (Supplementary Fig. S6) showed aromatic carbon peaks arising from benzene and thiophene rings occurring from 115 to 140 ppm. The peaks assigned to the acetylene carbon atoms linked to the benzene and thiophene rings were observed at 85 and 96 ppm, respectively. The intense peak observed at 155 ppm was assigned to the fluorine-substituted aromatic carbon atoms. This peak was not observed in the spectrum of the polymer obtained from 2,2′-(1,4-phenylene)bis(5-bromothiophene) and TEB (the molar ratio = 0.8:1) in toluene/DMF (1:1 v/v) (Supplementary Fig. S6). In the Fourier transform infrared (FT-IR) spectrum of **MP-0.8**, the peak at 3280 cm^–1^, assignable to the C–H stretching vibration of the ethynyl groups in the terminal **TEB** units, was very weak (Supplementary Fig. S7), indicating that most of the ethynyl groups of the **TEB** molecules participated in the polymerization reaction, as was expected. Polymer **MP-0.8** was thermally stable up to 300 °C (Supplementary Fig. S8) as measured using thermogravimetric analysis (TGA).

### Microstructure development

**Figure 4:**
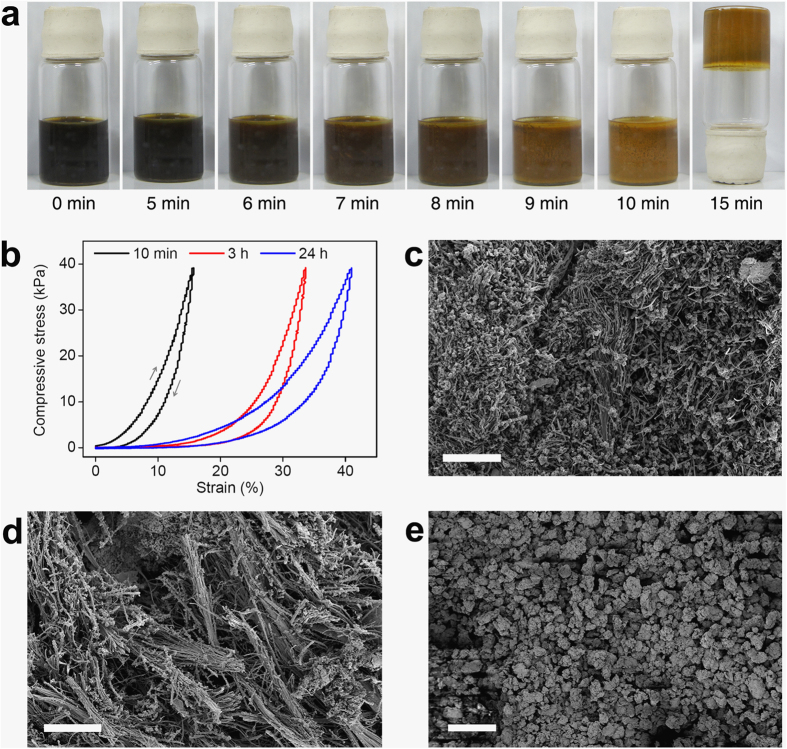
Effect of the reaction time on MP-0.8. (**a**) Time-dependent images of a reaction mixture of **PBT-Br** and **TEB** (molar ratio = 0.8:1) in toluene/DMF (1:1, v/v) on a hotplate. (**b**) Strain–stress curves of the samples obtained after different reaction times. SEM images of the samples obtained after a reaction time of (**c**) 10 min and (**d**) 3 h (scale bars = 40 μm). (**e**) SEM image of **MP-lin** (scale bar = 40 μm).

To follow the development of the tubular structures, we investigated the morphology of the products that formed in the early stages of the reaction. Figure 4a shows time-dependent images of the reaction products of **PBT-Br** and **TEB** (molar ratio = 0.8:1) in toluene/DMF (1:1, v/v). After a reaction time of 5 min at 100 °C, tiny particles precipitated out of the dark solution in which monomers and catalysts were fully dissolved. Soon after, a brown monolith began to form, filling the reaction vial. The reaction mixture became immobile within 15 min, and no visible flow occurred when the vial was turned upside down.

A monolithic solid (**MP-0.8–10 min**) isolated after a reaction time of 10 min was slightly compressible (Fig. 4b). Tubular entities, together with spherical particles, were observed in the SEM image of **MP-0.8–10 min**, suggesting that tubular structures formed at an early stage of the reaction (Fig. 4c). The microstructures of all the polymers obtained after a reaction time of 3 h appeared similar in their SEM images (Fig. 4d), but the compressibility of the polymers increased with increasing reaction time (Fig. 4b). The solid state ^13^C NMR spectrum of **MP-0.8–3 h** was similar to that of **MP-0.8** (Supplementary Fig. S9).

Tubular fibres and their bundles are intriguing morphologies that are not expected in polymers prepared from a cross-coupling reaction of multifunctional monomers. There have been a few reports on the formation of tubular structures of cross-linked polymers^33^,^34^,^35^,^36^, but the mechanism is not fully understood. The formation of tubular shapes is most likely induced by step reactions in combination with the structured self-assembly of the reactants. Presumably, linear oligomers having reactive ethynyl groups on each repeating unit are produced first from the cross-coupling reaction between two of the three ethynyl groups of **TEB** and two C–Br groups of **PBT-Br**. These develop into tubular structures on further reaction with monomers or oligomers. The reactivity of the soluble oligomers with twisted structures seems to play an important role in this process. **MP-lin** synthesized from **PBT-Br** and 1,3-diethynylbenzene instead of **TEB** under the same reaction conditions as those for **MP-0.8** does not form any tubular structures, as observed in the SEM image shown in Fig. 4e. Both monomers, **PBT-Br** and 1,3-diethynylbenzene, have a bifunctionality, and therefore, the resulting polymers should have linear structures.

### Origin of the compressibility

**Figure 5:**
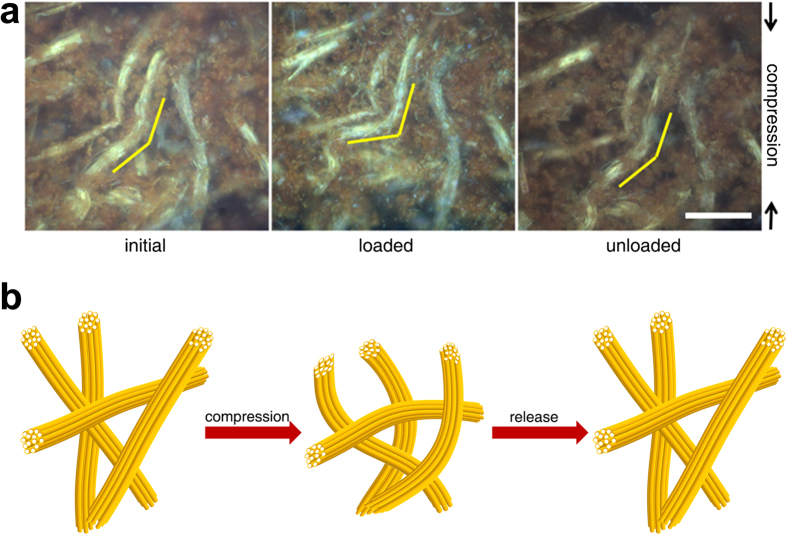
Mechanism of the compressibility of MP-0.8. (**a**) Optical microscope images of **MP-0.8** taken under loaded and unloaded conditions (scale bar = 50 μm). (**b**) Schematic drawing of the mechanism of the compressibility of **MP-0.8**.

To understand the origin of the compressibility, the change in microstructure of **MP-0.8** under stress was investigated using an optical microscope. Although we were unable to distinguish individual fibres because of the limited resolution of the optical microscope, some fibre bundles with lengths of tens of micrometres were clearly observed under the optical microscope (Fig. 5a). The polymer monolith resembled a non-woven fabric, where fibrous units are randomly entangled to form a monolith. The change in morphology of the polymer was imaged while a compressive stress was applied. As denoted by the yellow lines in Fig. 5a, the bundles were bent in the direction perpendicular to the direction of compressive stress. The initial structure was restored upon release of the stress, which suggested that the mechanism of the compressibility was linked to the elastic buckling of the bundled tubular assemblies. Individual tubes would not have enough durability to maintain their structure under compressive stress. However, through bundling they are able to withstand the load because the assembled tubes are able to support each other and distribute the loaded stress^37^,^38^. This mechanism is shown schematically in Fig. 5b.

### Absorption properties

**MP**s are expected to show a high absorption capacity with selectivity towards hydrophobic liquids because of their aromatic hydrocarbon structures and microporosities^10^,^11^,^12^,^39^. Materials with such properties have potential applications in environmental pollution control, such as for marine oil spill recovery. Sorbent materials with compressibility will be particularly useful when the sorbents or sorbed substances need to be recycled.

**Figure 6:**
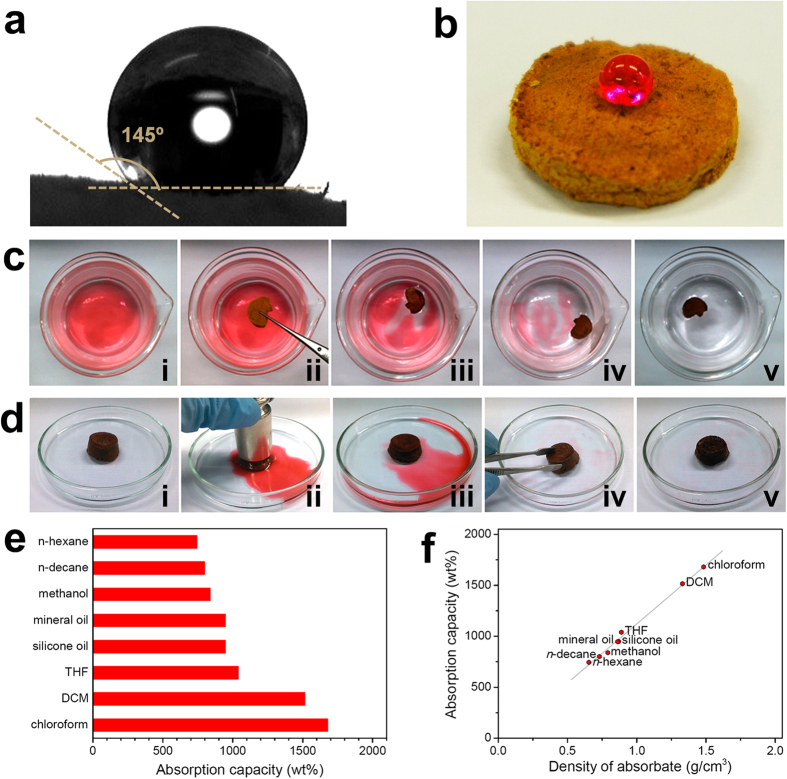
Hydrophobicity and absorption properties of MP-0.8. (**a**) The water contact angle measured on the surface of **MP-0.8**. (**b**) A droplet of water dyed with Rhodamine B on the surface of **MP-0.8**. (**c**) Removal of *n*-decane dyed with Oil Red O floating on water using **MP-0.8**. (**d**) Release of absorbed *n*-decane by compression and repeated absorption using **MP-0.8**. (**e**) Absorption capacity of **MP-0.8** for various liquids, and (**f**) their correlation with the absorbate density.

As shown in Fig. 6a,b, the surface of **MP-0.8** was very hydrophobic with a water contact angle of 145° 40. The selective absorption of a hydrophobic pollutant over water by **MP-0.8** was examined. A polluted aqueous sample was prepared by mixing water with *n*-decane dyed with Oil Red O for visualization. When a sample of **MP-0.8** was added to the solution, *n*-decane was selectively removed within a period of tens of seconds (Fig. 6c). The *n*-decane was released by simple compression and then reabsorbed by **MP-0.8** (Fig. 6d), demonstrating the possibility of recycling the polymer on use. The absorption properties of **MP-0.8** for various organic liquids were also investigated. **MP-0.8** could absorb 7–17 times its weight in liquid (Fig. 6e). There was a linear correlation between the absorption capacity of **MP-0.8** and the density of an absorbate (Fig. 6f), suggesting that the absorption capacity was dependent on the accessible pore volume.

Microporous materials can absorb and store small molecules more efficiently than mesoporous or macroporous materials can. For this reason, there has been extensive research into the application of microporous polymers for the removal of toxic chemicals in the air and in solutions^28^,^39^,^41^. We examined the possible use of **MP-0.8** for the removal of small molecules from a solution and their subsequent release by compression. Sudan I was chosen as a test molecule, which has been used as a food colouring, but now its usage is banned because of its genotoxic and carcinogenic properties^42^.

**Figure 7:**
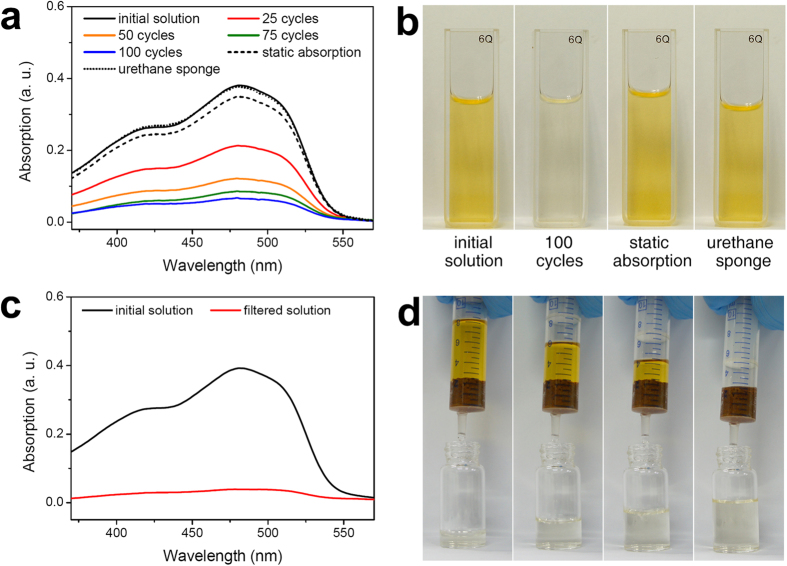
Efficient dye removal using MP-0.8. (**a**) UV–Vis spectra of Sudan I solutions (initial concentration = 5.0 × 10^–5^ M in ethanol) measured after removing a dye by **MP-0.8** (0, 25, 50, 75, and 100 cycles of compression and release, and a static absorption for 10 min) and by a urethane sponge (100 cycles of compression and release) (black circles). (**b**) Photographs of the above solutions. (**c**) UV–Vis spectra of the Sudan I stock solution and a filtered solution. (**d**) Sequential photographs showing Sudan I dye capture using an **MP-0.8** syringe filter.

A sample of **MP-0.8** (50 mg) was immersed in 5 mL of a solution of Sudan I in ethanol (5.0 × 10^–5^ M) and manually compressed and released *in situ* at a rate of about 5 s per cycle. The UV–Vis absorption of the solution was monitored every 25 cycles. Figure 7a shows that a gradual decrease in the absorption intensity of Sudan I in the solution was observed. The yellowish solution became almost colourless after 100 cycles, indicating that Sudan I had been removed by the polymer (Fig. 7b). In contrast, when a static absorption experiment was carried out by immersing the same mass of **MP-0.8** in 5 mL of the Sudan I solution (5.0 × 10^–5^ M) for 10 min^43^, the UV–Vis spectrum of the resulting solution showed only a small decrease in the concentration of Sudan I. It appears that the repeated compression and release facilitated interfacial contact between Sudan I in the solution and the **MP-0.8** with a resulting enhancement of the absorption performance of the polymer. The microporosity of **MP-0.8** could also be an important factor influencing the absorption performance, as it would impede the release of any adsorbed Sudan I during compression. To verify this proposition, the same tests were performed using a commercially available macroporous urethane sponge (*V*_*tot*_ = 0.168 cm^3^g^–1^ at *p*/*p*_*0*_ = 0.99 and *V*_*micro*_ = 6.34 × 10^–3^ cm^3^g^–1^ at *p*/*p*_*0*_ = 0.10; Supplementary Fig. S10). The urethane sponge was immersed statically in a Sudan I solution for 10 min and was also compressed and released 100 times. The UV–Vis spectra of the solutions measured after the tests were almost the same, and were similar to the spectrum of the initial solution, indicating that Sudan I was barely absorbed by the urethane sponge.

**MP-0.8** could be also used as a syringe filter for more facile and rapid removal of a dye^44^ (Fig. 7c,d). Due to its monolithic character, **MP-0.8** was fabricated into a shape that fitted into the inner diameter of a syringe. We placed a Sudan I solution into a syringe whose outlet was blocked with **MP-0.8** and then pressed the solution out through the material. The yellow Sudan I solution turned colourless as it passed through the **MP-0.8**. The UV–Vis spectrum of the filtrate showed that most of the dye molecules were removed in this process (Fig. 7c).

### Carbonization

**Figure 8:**
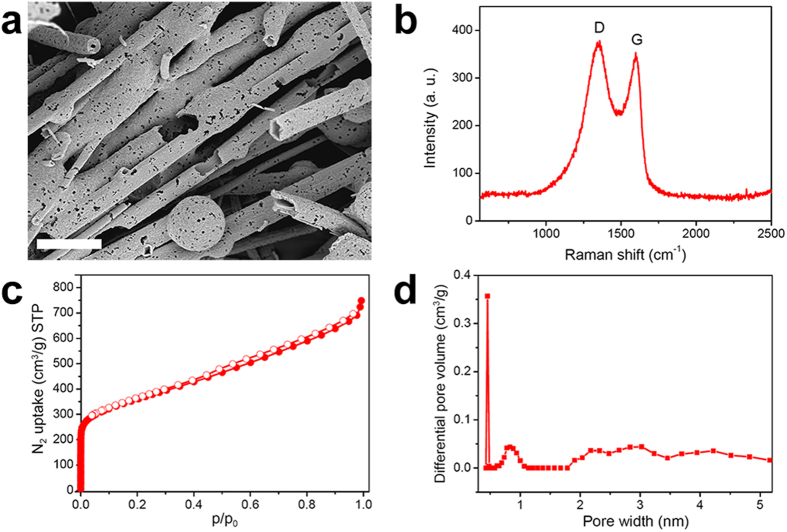
Carbonization of MP-0.8. (**a**) SEM image (scale bar = 2 μm) and (**b**) Raman spectrum of **MP-0.8-C**. (**c**) N_2_ adsorption–desorption isotherms of **MP-0.8-C** measured at 77 K. (**d**) NL-DFT pore size distribution of **MP-0.8-C**.

**MP**s with tubular structures are potential precursors of structured carbon materials^35^. **MP-0.8** was carbonized at 800 °C under inert N_2_ gas to form **MP-0.8-C** with a char yield of 47.6%. An SEM study showed that the tubular structure of **MP-0.8** was maintained after pyrolysis (Fig. 8a). Newly formed holes observed on the tube surfaces indicated that some organic entities were vaporized during the pyrolysis. Raman spectroscopy showed a G band occurring at 1598 cm^–1^ together with a D band occurring at 1355 cm^–1^ (Fig. 8b), indicating that **MP-0.8-C** was partially graphitic. The BET surface area increased markedly to 1288 m^2^/g after carbonization (Fig. 8c). The NL-DFT pore size distribution showed a generation of ultra-micropores with sizes around 0.45 nm (Fig. 8d)^45^. **MP-0.8-C** did not exhibit a compressible and monolithic character.

## Discussion

We have observed the formation of compressible monolithic polymers, **MP**s, from a cross-coupling reaction of multifunctional aromatic compounds. A microscope study revealed that the polymers consisted mainly of tubes and their bundles, together with spherical particles. The polymer morphology was influenced by the experimental conditions, such as the monomer feed ratio and solvent used. Although the mechanism for the development of these intriguing **MP** morphologies requires further research, our study suggests that tubular shapes are most likely formed by step reactions in combination with structured self-assembly of the reactants in the reaction mixture, such as monomers, linear oligomers, and branched polymers having reactive ethynyl groups. Regarding the observation of compressibility of the polymers, the bundled tubular structures appear to be responsible for this *via* elastic buckling under stress. As the tube bundles are rigid and are strong enough to endure compressive stress, they did not undergo structural destruction under load and recovered their original macroscopic shape once the stress was removed. The polymers showed a degree of microporosity, which was suitable for the removal of toxic chemicals from the environment. In particular, the compressibility and monolithic character of these polymers allowed for the facile release of adsorbed chemicals by applying pressure and thereby imparting a good recyclability. We believe that our discovery of microporous polymers with these unprecedented properties will contribute greatly to research into soft sorbent materials and to an increase in their applications.

## Methods

### Materials

All chemicals, reagents, and solvents were purchased from Sigma-Aldrich, Tokyo Chemical Industry, or Junsei Chemical and used without any further purification. Urethane sponge was obtained from a product manufactured by 3 M and washed with ethanol and acetone before using.

### Measurements

^1^H NMR spectra were recorded on a Bruker Advance 300 spectrometer (300 MHz). ^13^C NMR spectra were recorded on a Jeol JNM-LA400 (400 MHz). Solid-state ^13^C NMR spectra were recorded on a Bruker Avance 400 WB spectrometer (100 MHz) equipped with a CP/MAS probe. Elemental analyses were performed using a Flash EA 1112 elemental analyzer. Matrix-assisted laser desorption ionization-time of flight mass spectrum (MALDI-TOF/MS) was recorded on a MALDI TOF-TOF 5800 System. TGA were performed on a TA modulated TGA2050 with a heating rate of 10 °C/min under nitrogen. The compression test was performed on a KES-FB3 automatic compression tester. SEM images were obtained by Carl Zeiss SUPRA 55 VP. TEM images were obtained by a Carl Zeiss LIBRA 120 operating at 120 kV. TEM samples were dispersed in ethanol and a drop of the mixture was placed on a carbon-coated copper TEM grid. EDS elemental maps were obtained using an Oxford instrument X-Max^N^ detector and analyzed with AZtecEnergy EDS analysis. Optical Microscope images were obtained using an Olympus BX51. PXRD patterns were obtained using a New D8 Advance (Cu Kα radiation, λ = 1.54 Å). For a monomer, FT-IR measurement was made by a PERKIN ELMER Spectrum GX I using a KBr pellet. For polymers, FT-IR spectra were recorded on a Thermo Scientific Nicolet 6700 FT-IR spectrometer using attenuated total reflectance (window ZnSe/diamond). N_2_ uptake amounts were measured by a Belsorp-Max (BEL Japan, Inc.) apparatus. UV-Vis spectra were obtained with the use of a Sinco S-3150 spectrometer. The water contact angle was measured using a KSV CAM 101-Optical Contact Angle and Surface Tension Meter. Raman spectroscopy was conducted using a RAMANplus confocal laser Raman microscope (Nanophoton).

### Synthesis of 2,2′-(2,5-difluoro-1,4-phenylene)bisthiophene (PBT)

To a solution of 1,4-dibromo-2,5-difluorobenzene (1.50 g, 5.52 mmol) and Pd(PPh_3_)_4_ (255 mg, 0.221 mmol) in DMF (50 mL), was added 2-(tributylstannyl)thiophene (4.94 g, 13.2 mmol). After stirring at 100 °C in darkness for 24 h, the mixture was cooled down to room temperature, poured into distilled water and extracted with ethyl acetate. The organic layer was dried with MgSO_4_, filtered and the solvent was evaporated under reduced pressure. Remaining solid was recrystallized (tetrahydrofuran/ethanol, 1:9) to give colorless solid (998 mg, 65%). ^1^H NMR (300 MHz, CDCl_3_): δ 7.51 (d, J = 3.3 Hz, 2H), 7.44 (m, 4H), 7.15 (t, 2H); ^13^C NMR (400 MHz, THF-d^8^): δ 157.28, 154.75, 136.41, 128.65, 128.09, 123.02, 116.27; Analysis (calcd, found for C_14_H_8_F_2_S_2_): C (60.41, 60.69), H (2.90, 2.78), S (23.04, 23.07).

### Synthesis of 2,2′-(2,5-difluoro-1,4-phenylene)bis(5-bromothiophene) (PBT-Br)

To a solution of 2,2′-(2,5-difluoro-1,4-phenylene)bisthiophene (1.16 g, 4.17 mmol) in DMF (50 mL), was added *N*-bromosuccinimide (1.49 g, 8.37 mmol). After stirring at room temperature in darkness for 12 h, the mixture was poured into distilled water and extracted with ethyl acetate. The organic layer was dried with MgSO_4_, filtered and the solvent was evaporated under reduced pressure. Remaining solid was recrystallized (tetrahydrofuran/ethanol, 1:9) to give colorless solid (1.56 g, 86%). ^1^H NMR (300 MHz, CDCl_3_): δ 7.34 (t, 2H), 7.24 (d, J = 3.9 Hz, 2H), 7.09 (d, J = 3.9 Hz, 2H); ^13^C NMR (400 MHz, THF-d^8^): δ 157.15, 154.66, 137.81, 131.87, 128.32, 122.65, 115.77; Analysis (calcd, found for C_14_H_6_Br_2_F_2_S_2_): C (38.55, 38.72), H (1.39, 1.26), S (14.70, 14.76). FT-IR (KBr, cm^−1^): 3090, 1741, 1549, 1487, 1407, 1278, 1171, 873, 788, 487, 440. MALDI-TOF/MS spectrum is shown in Supplementary Fig. S11.

### General procedure for polymerization by Sonogashira-Hagihara coupling reactions

A typical procedure was as follows: **PBT-Br** (300 mg, 0.688 mmol), **TEB** (129 mg, 0.859 mmol), PdCl_2_(PPh_3_)_2_ (35.0 mg, 49.9 μmol), and CuI (11.5 mg, 60.4 μmol) were dissolved in a co-solvent of toluene (2.5 mL) and DMF (2.5 mL) and the mixture was heated up with stirring. At the moment the temperature reached 50 °C, triethylamine (TEA, 2.5 mL) was added dropwise and the reaction proceeded at 100 °C for 24 h. The produced polymer monolith was taken out of the reaction vessel and washed with methanol, water, tetrahydrofuran, and acetone. After Soxhlet extraction with methanol and drying *in vacuo* at 120 °C, the polymer (**MP-0.8**) was obtained as a light brown monolith. FT-IR (cm^−1^): 3280, 3069, 1681, 1581, 1496, 1456, 1175, 869, 799, 678. Analysis (found): C (68.94), H (2.99), S (11.22).

## Additional Information

**How to cite this article**: Lim, Y. *et al.* Compressible and monolithic microporous polymer sponges prepared *via* one-pot synthesis. *Sci. Rep.* **5**, 15957; doi: 10.1038/srep15957 (2015).

## Supplementary Material

Supplementary Information

Supplementary Movie S1
