# Supplementary material for: Compressible and monolithic microporous polymer sponges prepared via one-pot synthesis
Source: Sci Rep. 2015 Nov 4;5:15957. doi: 10.1038/srep15957 (PMC4632124; doi:10.1038/srep15957)
Supplement: Supplementary Information [file srep15957-s1.pdf]

Supplementary Information

Compressible and monolithic microporous polymer sponges prepared via one-pot synthesis

Yoonbin Lim, Min Chul Cha, and Ji Young Chang[*]

Department of Materials Science and Engineering

College of Engineering, Seoul National University

Seoul 151-744 (Korea)

E-mail: jichang@snu.ac.kr

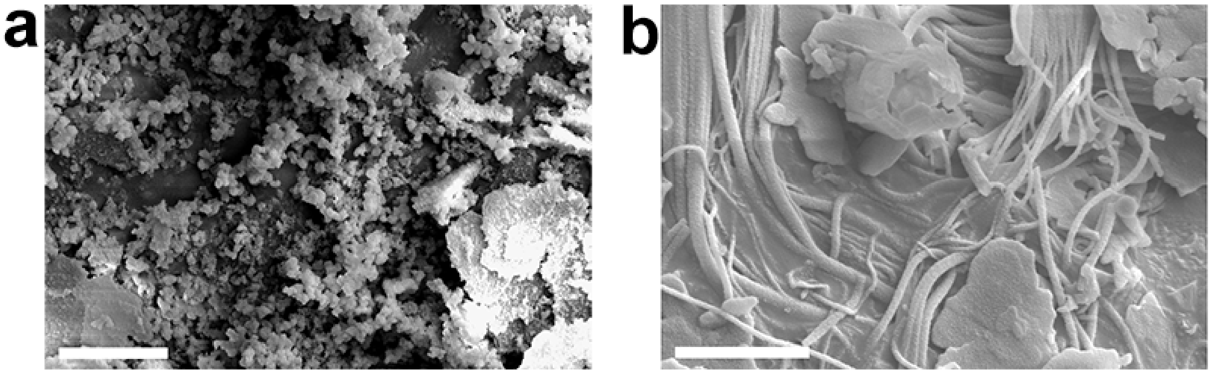

Supplementary Figure S1. SEM images of the polymers. SEM images of the polymers obtained by the reaction of 2,2'-(1,4-phenylene)bis(5-bromothiophene) and TEB (the molar ratio = 0.8:1) in toluene/DMF (1:1 v/v) (a) and in toluene (b) at 100 ^{o}C for 24 h (scale bar = 5 μm).

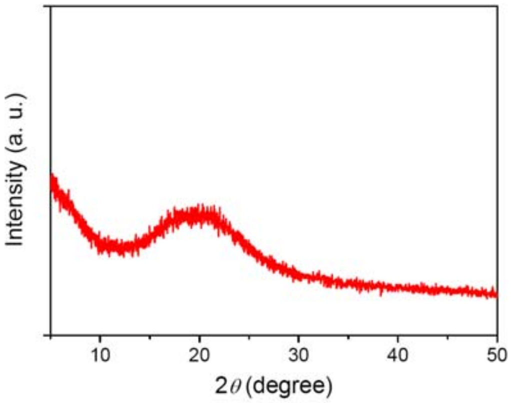

Supplementary Figure S2. PXRD measurement. PXRD pattern of **MP-0.8**.

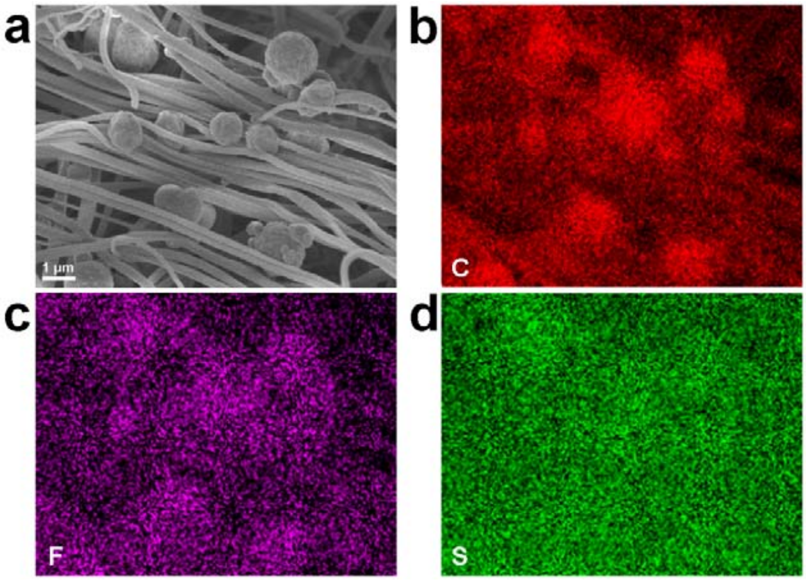

Supplementary Figure S3. EDS measurement. (a) SEM image and EDS elemental maps of (b) carbon, (c) fluorine, and (d) sulfur for **MP-0.8**.

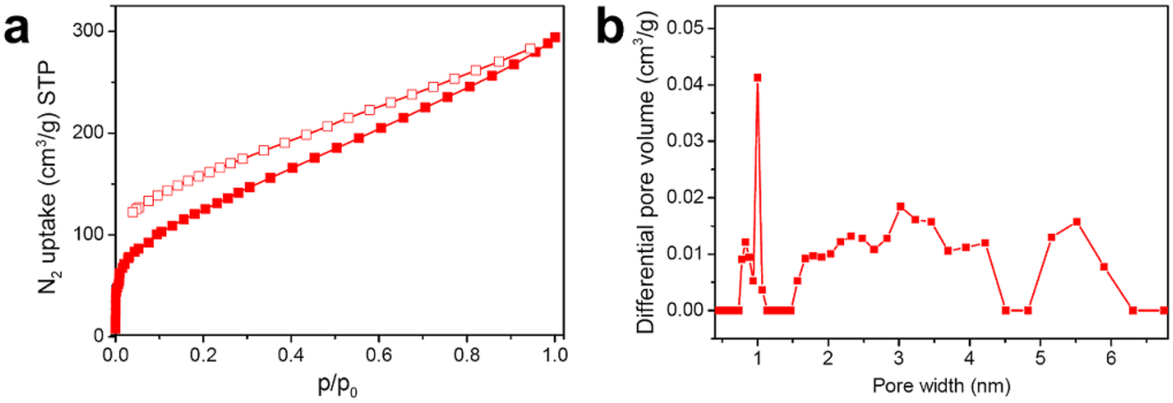

Supplementary Figure S4. Microporosity of MP-0.8. (a) N_2 adsorption–desorption isotherms of **MP-0.8** measured at 77 K. **(b)** NL-DFT pore size distribution of **MP-0.8**. The total pore volume and micropore volume of **MP-0.8** were 0.454 cm^3g^{-1} at p/p_0 = 0.99 and 0.157 cm^3g^{-1} at p/p_0 = 0.10, respectively.

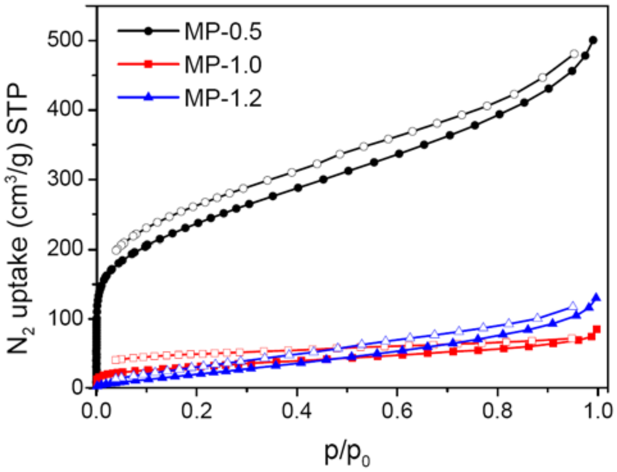

Supplementary Figure S5. Microporosities of MPs. N_2 adsorption-desorption isotherms of **MP-0.5**, **MP-1.0**, and **MP-1.2**.

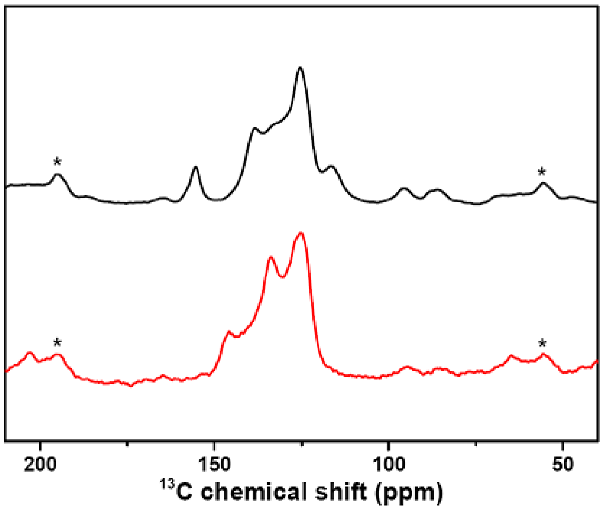

Supplementary Figure S6. NMR measurement. Solid state ^{13}C CP/MAS NMR spectrum of **MP-0.8** (black line) and the polymer obtained by the reaction of 2,2'-(1,4-phenylene)bis(5-bromothiophene) and TEB (the molar ratio = 0.8:1) in toluene/DMF (1:1 v/v) at 100 °C for 24 h (red line).

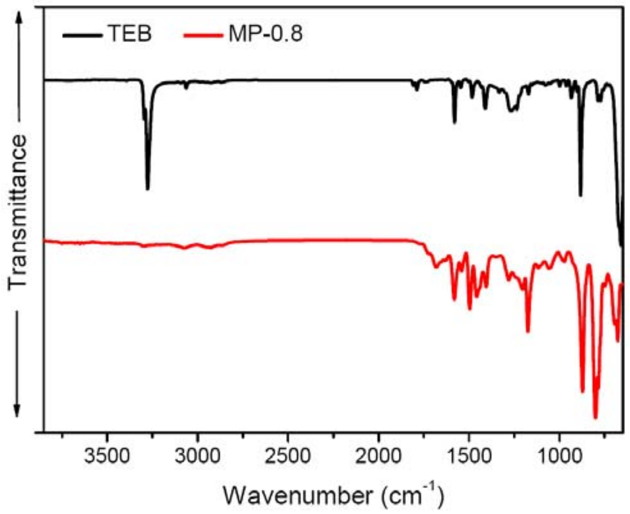

Supplementary Figure S7. FT-IR measurement. FT-IR spectra of **TEB** and **MP-0.8**. For the monomer, the FT-IR spectrum was recorded on a PERKIN ELMER Spectrum GX I using a KBr pellet. For the polymer, the FT-IR spectrum was recorded on a Thermo Scientific Nicolet 6700 FT-IR spectrometer using attenuated total reflectance (window ZnSe/diamond).

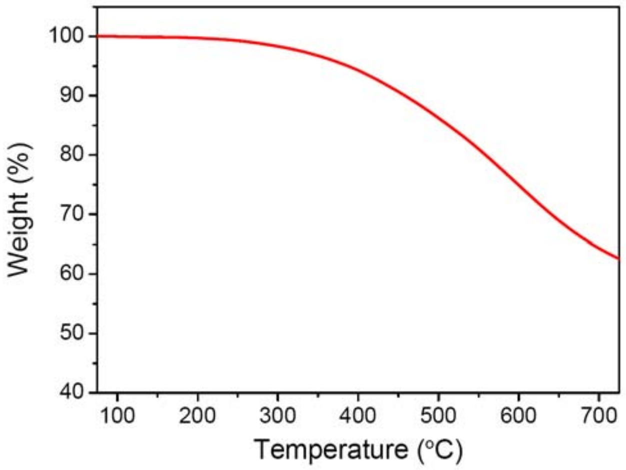

Supplementary Figure S8. TGA measurement. TGA curve of **MP-0.8**.

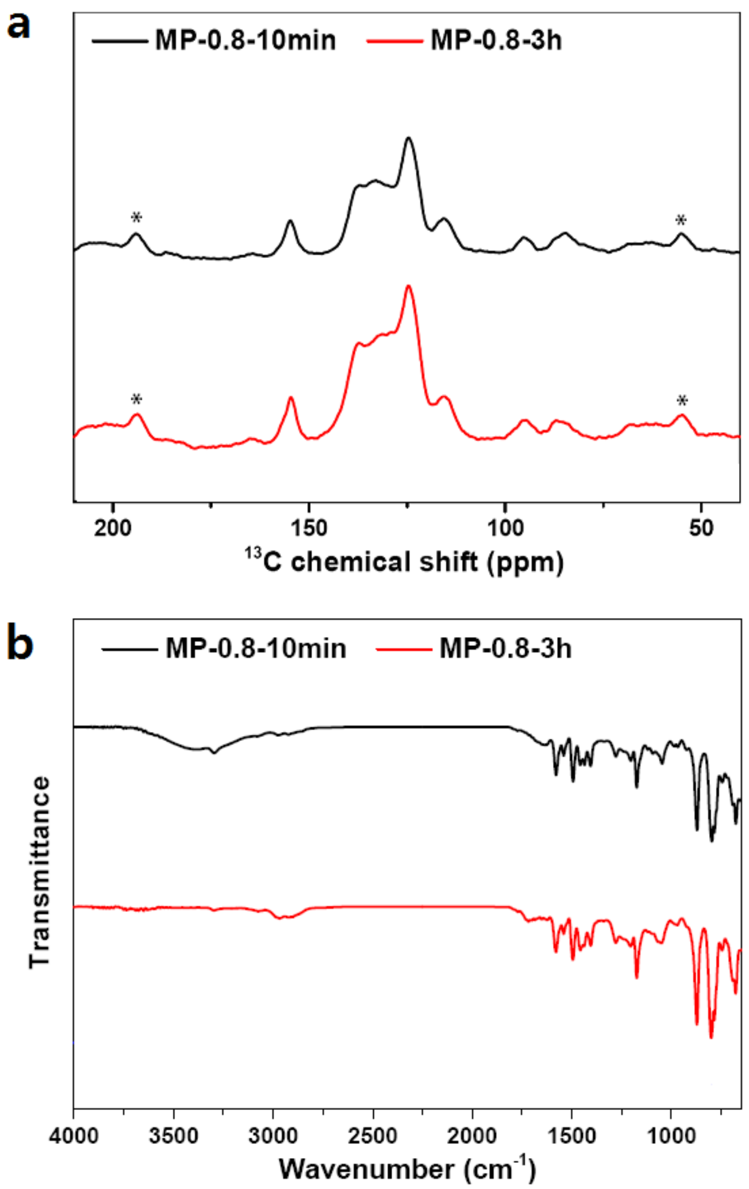

c

	carbon	hydrogen	sulfur
MP-0.8-10min	69.39	2.65	12.23
MP-0.8-3h	69.33	2.70	11.84

Supplementary Figure S9. Characterization of the polymers. (a) Solid state ^{13}C CP/MAS NMR spectra, (b) FT-IR spectra and (c) elemental analysis results for **MP-0.8-10min** and **MP-0.8-3h**.

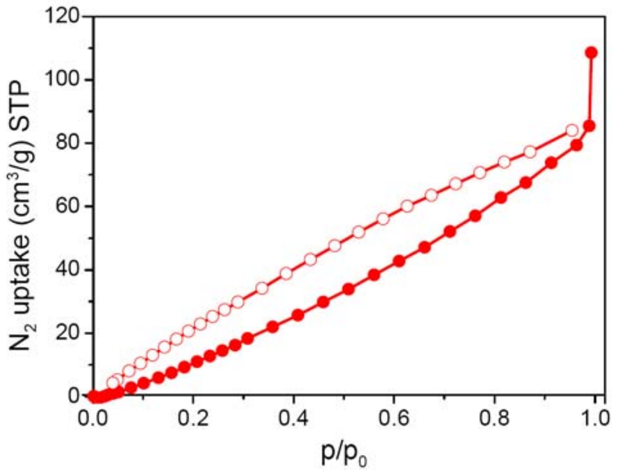

Supplementary Figure S10. Macroporosity of urethane sponge.

Supplementary Figure S10. Macroporosity of urethane sponge. N_2 adsorption-desorption isotherms of the urethane sponge used in this study. V_{tot} = 0.168 cm^{3}g^{-1} at p/p_0 = 0.99 and V_{micro} = 6.34 × 10^{-3} cm^{3}g^{-1} at p/p_0 = 0.10.

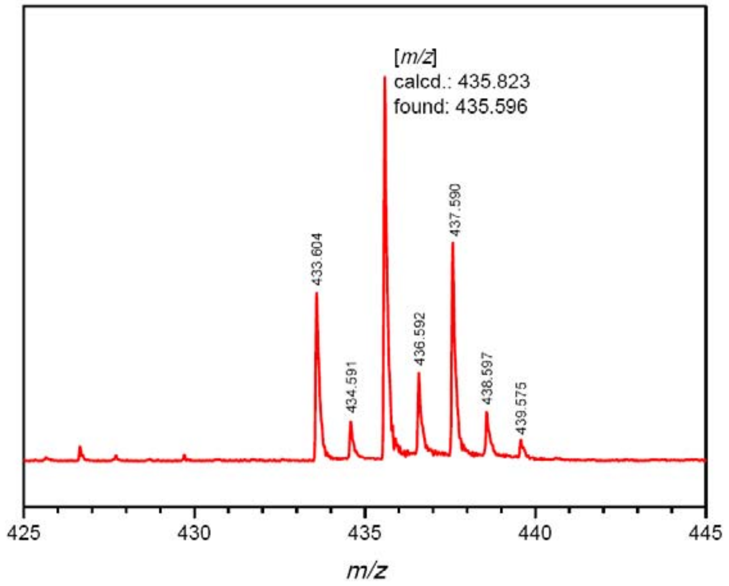

Supplementary Figure S11. MALDI-TOF MS spectrum of PBT-Br. Calculated [m/z]: 435.823 (100%), 433.825 (51%), 437.820 (49%), 436.826 (10%), 438.824 (7%), 434.828 (4%), 439.816 (4%); Found: 435.596 (100%), 433.604 (44%), 437.590 (57%), 436.592 (23%), 438.597 (13%), 434.591 (11%), 439.816 (5%).

2,2'-(1,4-Phenylene)bisthiophene. This compound was prepared following the procedure for the preparation of **PBT** in 72% yield. ^{1}H NMR (300 MHz, $CDCl_3$, ppm): δ 7.62 (s, 4H), 7.34 (d, J = 3.6 Hz, 2H), 7.29 (d, J = 5.1 Hz, 2H), 7.10 (t, 2H). ^{13}C NMR (400 MHz, THF-d^8, ppm): δ 144.73, 134.65, 129.05, 127.04, 125.89, 124.20. Analysis (calcd, found for $C_{14}H_{10}S_2$): C (69.38, 69.35), H (4.16, 4.19), S (26.46, 26.43).

Synthesis of 2,2'-(1,4-phenylene)bis(5-bromothiophene). This compound was prepared following the procedure for the preparation of **PBT-Br** in 93% yield. ^{1}H NMR (300 MHz, $CDCl_3$, ppm): δ 7.51 (s, 4H), 7.08 (d, J = 3.9 Hz, 2H), 7.05 (d, J = 3.9 Hz, 2H). The collection of ^{13}C NMR data was precluded due to sparing solubility. Analysis (calcd, found for $C_{14}H_8Br_2S_2$): C (42.02, 42.19), H (2.02, 2.04), S (16.03, 16.15).
